# Mixed Pt-Ni Halide Perovskites for Photovoltaic Application

**DOI:** 10.3390/ma17246196

**Published:** 2024-12-18

**Authors:** Huilong Liu, Rubaiya Murshed, Shubhra Bansal

**Affiliations:** 1 School of Mechanical Engineering, Purdue University, West Lafayette, IN 47907, USA; liu3601@purdue.edu; 2 Department of Mechanical Engineering, Embry-Riddle Aeronautical University, Daytona Beach, FL 32114, USA; murshed.rubaiya21@gmail.com; 3 Department of Mechanical Engineering, University of Nevada Las Vegas, Las Vegas, NV 89154, USA; 4 School of Materials Engineering, Purdue University, West Lafayette, IN 47907, USA

**Keywords:** perovskite solar cells, Cs_2_PtI_6_, lead-free, mixed Pt-Ni, cost analysis, thermal stability

## Abstract

Cs_2_PtI_6_ is a promising photoabsorber with a direct bandgap of 1.4 eV and a high carrier lifetime; however, the cost of Pt inhibits its commercial viability. Here, we performed a cost analysis and experimentally explored the effect of replacing Pt with earth-abundant Ni in solution-processed Cs(Pt_x_Ni_1−x_)(I,Cl)_3_ thin films on the properties and stability of the perovskite material. Films fabricated with CsI and PtI_2_ precursors result in a perovskite phase with a bandgap of 2.13 eV which transitions into stable Cs_2_PtI_6_ with a bandgap of 1.6 eV upon annealing. The complete substitution of PtI_2_ in films with CsI + NiCl_2_ precursors results in a wider bandgap of 2.35 eV and SEM shows two phases—a rod-like structure identified as CsNi(I,Cl)_3_ and residual white particles of CsI, also confirmed by XRD and Raman spectra. Upon extended thermal annealing, the bandgap reduces to 1.65 eV and transforms to CsNiCl_3_ with a peak shift to higher 2-theta. The partial substitution of PtI_2_ with NiCl_2_ in mixed 50-50 Pt-Ni-based films produces a bandgap of 1.9 eV, exhibiting a phase of Cs(Pt,Ni)(I,Cl)_3_ composition. A similar bandgap of 1.85 eV and the same diffraction pattern with improved crystallinity is observed after 100 h of annealing, confirming the formation of a stable mixed Pt-Ni phase.

## 1. Introduction

In recent years, perovskite photovoltaic technology has offered enormous viability and dimensionality in solar cell research. Perovskite, as a light-harvesting active layer, has generated a remarkable development in device efficiency of 25.6% in the single-junction solar cell, and over 33% in perovskite/silicon tandem solar cells [1,2]. Also, the all-perovskite tandem solar cell is showing great potential in device performance and has thus far achieved a power conversion efficiency (PCE) of 26.4% with a wide bandgap (WBG) FA_0.8_Cs_0.2_Pb(I_0.62_Br_0.38_)_3_ perovskite as the top subcell (1.8 eV) and a thermally mixed Sn/Pb narrow bandgap (NBG) FA_0.7_MA_0.3_Pb_0.5_Sn_0.5_I_3_ perovskite as the bottom subcell (1.2 eV) [3]. Transitioning photovoltaic technology from the laboratory to commercial products, high power conversion efficiency, low cost, long lifetime, and low toxicity are some of the critical factors to consider during material selection [4]. Pb-halide perovskites have been the most studied compositions in next-generation photovoltaics due to their excellent optoelectronic properties, such as the highest power conversion efficiency (PCE) and ideal bandgap [5,6,7]. However, the practical relevance of these materials is hindered as they offer multifarious disadvantages, including toxicity, high water solubility and bioavailability, and thermodynamic instability of CH_3_NH_3_PbI_3_ in air [4,6,8,9]. To address the toxicity issue, Pb-free perovskite compounds have been the mainstay of perovskite research. Among the several alternative cations to Pb from the group-14 elements, the Sn-based perovskite absorbers are the widely studied alternative which are superior in achieving high efficiency due to their isoelectronic configuration of s^2^p^2^ similar to Pb and their smaller radius (1.35 Å and 1.49 Å in Sn^2+^ and Pb^2+^, respectively) [10,11]. Due to its smaller size than Pb, incorporating Sn in ASnPbX_3_ systems increases the tolerance factor and decreases the bandgap [12]. However, the high oxidation tendency from Sn^2+^ to Sn^4+^ upon exposure to air and the energy band level mismatch between the perovskite and the charge transport layers generate high recombination at the grain boundaries in Sn-based perovskites and hence a lower PCE than that of Pb-based perovskites [10,13]. Recently, transition metal-based double perovskite, Cs_2_PtI_6_, has emerged as another potential alternative to Pb-free perovskite owing to its experimentally produced direct narrow bandgap of 1.37 eV [14] and 1.4 eV [15]. Our previous work reported an atmospherically processable Cs_2_PtI_6_ perovskite device with an excellent absorption coefficient of 4 × 10^−5^ cm^−1^ and the highest efficiency of 13.88% [15]. The high V_oc_ comparable to a Pb-based perovskite device and the high minority carrier lifetime of over 2.8 μs with ethylene diamine (EDA) achieved in our study reveal Cs_2_PtI_6_ as a competitive Pb alternative for high-performance halide perovskite solar cells (HPSCs). Our theoretical investigation revealed key strategies, such as eliminating parasitic losses and optimizing band offset, to achieve a Cs_2_PtI_6_ photoabsorber with a PCE over 26% [16]. However, due to the high cost of Pt, the Cs_2_PtI_6_ perovskite is viable mostly as a model system [15]. Several other transition metals, such as copper (Cu), silver (Ag), nickel (Ni), and palladium (Pd), etc., have been reported in a few studies. Nag et al. studied inorganic double perovskite with monovalent Ag and trivalent Bi (i.e., Cs_2_AgBiX_6_), showing analogous optoelectronic properties similar to CsPbX_3_ with an eco-friendly nature and a long carrier lifetime [17]. However, it exhibits an indirect bandgap with low optical properties leading to a low power conversion efficiency [18,19]. Soni et al. [20] numerically studied several transition metal-based halide double Cs_2_ZSbX_6_ perovskites for photovoltaic applications, with Z = Ag and Cu. The bandgap reduction by replacing Ag (2.542 eV) with Cu (1.299 eV) in the B-site and X = Cl was attributed to the high absorption of incident photons in the broad optical spectrum within the framework of their DFT analysis [20]. Having high dielectric constants of about 5.33 and 6.30 for Cs_2_AgSbI_6_ and Cs_2_CuSbI_6_, respectively, and being optically active in the visible and ultraviolet regions, these materials can be productively utilized for optoelectronic devices [20]. CsNiCl_3_ and CsNiBr_3_ perovskites exhibiting a low electronic bandgap and dispersive band edges are expected to offer attractive photovoltaic characteristics [21]. The use of precious metals impedes the commercialization progress of perovskite solar cells. Non-precious transition metals are promising candidates for the counter electrode of perovskite solar cells owing to their cost–performance ratio. Low-cost non-precious transition metals are investigated in several studies to replace expensive metals, such as gold (Au) or silver (Ag), as counter electrode materials in perovskite solar cells. Wang et al. [22] prepared perovskite solar cells with transition metals, such as Cu and Ni, and presented a satisfactory performance with a power conversion efficiency of 13.04 and 12.18%, respectively, compared to that of 15.97% of the perovskite solar cell with a Ag counter electrode. Ni, having a very close work function (~5.04 eV) to that of Au (~5.1 eV), showed a power conversion efficiency (PCE) of 10.4%, comparable to devices with Au electrodes (11.6%) [23]. Cu-based perovskites are particularly advantageous because of their lower toxicity, magnetic properties [24], enhanced structural flexibility, and greater stability against light and humidity compared to Sn-based perovskites [25,26]. Several research groups have explored the use of Cu-based layered perovskites in the application of superconductors [25,27,28]. Cu, having a higher work function (~4.63 eV) than Ag (~4.23 eV), offers a higher voltage output [22]. Perovskite materials also show promise in gas sensor applications due to their unique electrical and catalytic properties [29,30]. Among various target gases, the detection of hydrocarbons is significantly important in various applications. For example, monitoring dissolved gases (CH_4_, C_2_H_4_, C_2_H_2_, CO, CO_2_, and H_2_) by gas-in-oil analysis in a transformer provides important information about transformer status [31,32]. Moreover, the detection and control of ethylene gas in agriculture are extremely important as emissions of this gas indicate the maturity state of fruits [33]. Ni-based perovskites are potential candidates for gas-sensing applications, as NiI_2_ exhibits a large impedance change in an ultra-low humidity environment [34]. Several research groups have developed lanthanum–transition metal perovskites, such as La-Co-based perovskite systems, in the application of catalytic oxidation and combustion [35,36,37]. However, not enough information is available in the literature on Ni-based perovskites for photovoltaic applications. For the A-site cations, formamidinium (FA), methyl ammonium (MA), and cesium (Cs) are considered the most preferable elements to form perovskite structures due to the preferred tolerance factor in the range of 0.8 to 1 [FAPbI_3_ (t~0.99), CsPbI_3_ (t~0.8) MAPbI_3_ (t~0.9)] [38,39]. CH_3_NH_3_I has a high decomposition rate into CH_3_I and NH_3_ at low temperatures [40], and the unstable photoactive black cubic phase of FA-based perovskites transitions into the photoinactive yellow phase at room temperature [41]. Cation-enabled perovskite black phase stabilization by partially incorporating inorganic Cs^+^ cations has been proven effective in enhancing the photo and moisture stability of perovskite [42]. The purpose of this study is to assess how partially replacing Pt in cesium platinum triiodide (CsPtI_3_) perovskite with different concentrations of earth-abundant and low-cost nickel (Ni) influences its crystallographic and optoelectronic properties. Considering a partial replacement of Pt^2+^ sites by Ni^2+^ should not cause severe lattice distortion due to the similar ionic radius of Ni^2+^ in comparison to Pt^2+^ (Ni^2+^ vs. Pt^2+^: 72 pm vs. 80 pm), and we assume Ni^2+^ could likely incorporate within the perovskite crystal lattice, given the high solid solubility of Pt and Ni. Cai et al. [43] performed first-principles calculations of halide perovskite-derived A_2_BX_6_ inorganic compounds to investigate the trends in bandgaps and energetic stability with chemical compositions, providing guidelines for the design of halide A_2_BX_6_ compounds for potential photovoltaic applications. According to this, perovskite compounds with X = I and B = Ni exhibit a large energy above the hull (*E_hull_*) (29 meV) and were not experimentally observed. All experimentally reported compounds have zero or small values of E_hull_, such as Cs_2_NiCl_6_ and Cs_2_PtI_6_ which have zero *E_hull_*. *E_hull_* is the difference between the formation energy of the compound and the energy on the convex hull in the phase diagram at the same composition. It describes the thermodynamic stability of a compound, and it can be expressed as follows:

$$E_{hull}=E_{compound}-E_{hull,stable}$$

where $E_{compound}$ is the formation energy of the compound and $E_{hull,stable}$ is the formation energy of the most stable phase (or combination of phases) at that composition. If $E_{hull}=0$, the compound lies on the convex hull and is thermodynamically stable. If $E_{hull}>0$, the compound is metalstable or unstable [44]. Replacing PtI_2_ with NiCl_2_ in varying molar ratios may enable the substitution of Pt with Ni in the mixed-metal perovskite, potentially leading to new methods for developing a more cost-effective system. To our best knowledge, this is the first study on the partial substitution of Pt by Ni performed experimentally for photovoltaic application.

## 2. Materials and Methods

### 2.1. Materials

Unless otherwise stated, all chemicals and materials were purchased and used on receipt. Cesium iodide (CsI), platinum (II) iodide (PtI_2_), nickel (II) chloride (NiCl_2_), dimethyl sulfoxide (DMSO), dimethylformamide (DMF), isopropanol (IPA), and acetone were purchased from Sigma Aldrich (St. Louis, MO, USA). The commercial FTO (fluorine-doped tin oxide) glass (Tec 10) was purchased from Ossila (Sheffield, UK).

### 2.2. Methods

**Figure 1:**
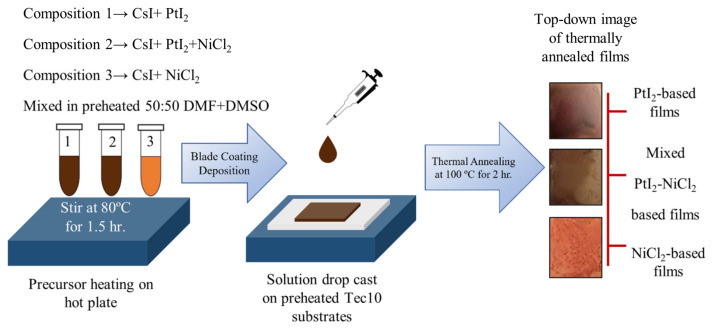
Atmospheric synthesis of PtI_2_, mixed PtI_2_-NiCl_2_, and NiCl_2_-based films in 50:50 DMF: DMSO via solution processing.

In this study, we chemically synthesized three different perovskite compositions, referred to as PtI_2_-based films, mixed PtI_2_-NiCl_2_-based films, and NiCl_2_-based films. All the films were fabricated via precursor-based solution processing under atmospheric conditions, as shown in Figure 1. The synthesis process of the 3 types of films is expected to be led by the following solid-state reactions, respectively:

CsI + PtI_2_ = CsPtI_3_, (1)

CsI + xPtI_2_ + yNiCl_2_ = Cs(Pt_x_, Ni_y_)(I, Cl)_3_, (2)

CsI + NiCl_2_ = CsNi(I_1_Cl_2_), (3)

where the x-to-y ratio of 50:50 has been used. The procedure and instruments for film fabrication and testing follow our previous works [15,45]. The precursor for the mixed PtI_2_-NiCl_2_-based films was prepared in a 0.25 M solution by the mixture of Cesium Iodide (0.06495 g), Platinum (II) Iodide (0.05611 g), and Nickel (II) Chloride (0.0162 g) with a molar ratio of 50:50 in 1 mL of 50%/50% volume mixture of dimethylformamide (DMF) and dimethyl sulfoxide (DMSO) solvent. We applied commercial FTO glass (Tec 10) as substrate for the thin-film processing. The FTO glass was cleaned via ultrasonication in a sequence of Alconox solution (15 min), deionized water (15 min), acetone (15 min), and IPA (30 min). Then, the substrates were dried before thin-film deposition. The precursor mixture was heated at 75 °C for 1.5 h, followed by drop-casting on the preheated Tec10 substrate. The doctor-blade coating technique was used to spread the solution over the preheated substrates. This step is performed in an atmospheric environment. Films were then annealed in a vacuum oven at −15 in Hg and 100 °C for 2 h. The same procedure was followed to fabricate the PtI_2_-based and NiCl_2_-based films. The thermally annealed PtI_2_-based films and mixed PtI_2_-NiCl_2_-based films were a dark reddish-black color, and the NiCl_2_-based films were an orange color. For stability testing, the films were exposed to a dark thermal annealing test at 100 °C for 100 h. All the solutes were purchased from Alfa-Aesar, Haverhill, MA, USA (Cesium Iodide, Alfa Aesar CAS: 7789-17-5; Platinum (II) Iodide, Alfa Aesar CAS: 7790-39-8; Nickel (II) Chloride, Alfa Aesar CAS: 7718-54-9), and solvents from Sigma-Aldrich, St. Louis, MO, USA (DMF, Sigma-Aldrich CAS: 68-12-2; DMSO, Sigma-Aldrich CAS: 67-68-5). The films were stored in a nitrogen-filled glove box before testing. X-ray diffraction spectroscopy (XRD) measurements were conducted on a Bruker diffractometer from Bruker Corporation Billerica, MA, USA, under ambient conditions using Cu K radiation. The EVA toolbox and Topas were used for XRD data analysis and phase identification. The Shimadzu UV-2600 spectrometer (Shimadzu, Carlsbad, CA, USA) was used to perform optical transmittance and reflectance measurements. The optical bandgap of the samples was determined by Tauc analysis. A JEOL JSM-5610 (JEOL, Tokyo, Japan) was used to perform Scanning Electron Microscopy (SEM) and Energy Dispersive X-ray Spectroscopy (EDS) analysis. Raman spectra were measured at room temperature using the customized Scope Foundry-based Raman spectrometer at Molecular Foundry, Berkeley National Laboratory. The microscope was in confocal geometry with 1800 g/mm grating and equipped with a silicon CCD. A continuous-wave 532 nm laser was used for excitation with 100 μW (0.366 mW/μm^2^), and a 532 nm long-wave pass filter was set in the output path.

## 3. Results and Discussion

### 3.1. Cost Analysis of Solutes for Perovskite Precursor and Encapsulation for HPSCs

This cost model determines the USD/watt of several perovskite material compositions, considering the molarity (M), absorber layer thickness (t), active cell area, and power conversion efficiencies (PCEs) from the respective literature. In this cost analysis, we compared the USD/watt value associated with the precursor solutes required to prepare these compositions and the additional cost of four different encapsulants. To estimate the cost, the cost for the precursor materials is based on the price of the chemicals listed on Sigma-Aldrich. Similarly, the cost of encapsulation materials, such as PET, is derived from the prices available on the vendor’s website (Table S3). The cost per gram (g) of solutes and properties of perovskite compositions are summarized in Tables S1 and S2, respectively. The molarity used in the literature for respective material compositions remains constant throughout this analysis. We also evaluated the USD/watt with the value of PCE (25.6%) and t (~2 µm) optimized to the value of the state-of-art FAPbI_3_ HPSCs [2]. The cost per unit volume is determined by the perovskite (product) volume, the proportional amount of reactants needed to achieve the product volume, and the cost of solutes per gram. The amount of perovskite, by moles, is determined using the known molecular mass (g/mol) and density (g/cm^3^) of the product as seen in Equation (4).

(4) $$\frac{Moles\;of\;Perovskite}{cm^{3}}\left[mol{/cm}^{3}\right]=\frac{Density\left[g/{cm}^{3}\right]}{Molar\;mass\left[g/mol\right]}$$

**Figure 2:**
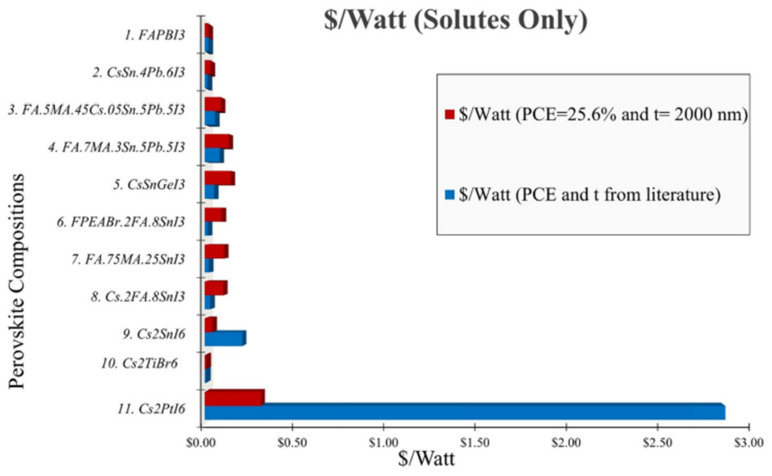
USD/Watt (solute) of various Pb and Pb-free perovskite compositions calculated with respect to the PCE and thickness reported in the corresponding literature (blue) and the highest PCE of 25.6% and thickness of 2000 nm reported for the Pb-based FAPbI_3_ perovskite (red). Figure S1 represents the USD/watt with the discrete effect of optimized PCE and absorber layer thickness.

Figure 2 is a graphical representation of the comparison of the USD/watt of the precursor solutes required to prepare several perovskite compositions considering two cost frameworks. The first cost framework (blue) represents the USD/watt when both the PCE and absorber layer thickness (t) are derived from the corresponding literature respective to each perovskite composition. The second cost framework (red) represents the USD/watt considering each perovskite composition matching both the PCE of 25.6% and t of 2000 nm as reported for the state-of-art FAPbI_3_ perovskite. By comparing these projected costs (red) with the actual cost framework (blue), it is clear that the current Pt-based perovskite exhibits a significantly lower efficiency and cost-effectiveness compared to other lead-based and lead-free perovskites. However, with proper optimization, Pt-based perovskite has the potential to achieve a cost efficiency comparable to that of other lead and lead-free perovskite.

It is important to note that the overall cost associated with perovskite products depends on many other several things, such as the precursor synthesis methods, processing conditions, film stability, and the use of additives and encapsulations, etc. Our current analysis of USD/watt only associates the cost of the solutes used for the corresponding precursor. According to Figure 2 (blue), the USD/watt of Cs_2_PtI_6_ is estimated to be ~144-times more expensive than that of FaPbI_3_ perovskite. The synergistic effect of a thicker absorber layer of 10,000 nm and the high cost of the PtI_4_ chemical compound (PtI_4_ = 226.55 USD/gm, PbI_2_ = 1.176 USD/gm) are responsible for the high USD/watt of its precursor. The distinct impact of the PCE and t on the cost modeling is presented in Figure S1. According to this, if the thickness (t) of the Pt-based composition remains constant at 10,000 nm and the PCE increases to 25.6%, the USD/watt is reduced to nearly half of its original value. by almost half the initial USD/watt value. Alternatively, if the PCE remains constant at 13.88% and t reduces to 2000 nm, the USD/watt of Cs_2_PtI_6_ reduces by almost five times the initial USD/watt value with the reported PCE and t. A thicker absorber layer requires more product and hence the t and cost of the solutes play a vital role in regulating the perovskite precursor cost. Our previous study numerically optimizes the thickness of several perovskite absorbers needed to reach the state-of-the-art PCE [16]. If both the parameters are optimized (PCE to 25.6% and t to 2000 nm), the USD/watt of Cs_2_PtI_6_ is estimated to be only ~15-times more expensive than the FaPbI_3_ perovskite, as indicated in Figure 2 (red), and reduces by almost nine times the initial USD/watt value with the reported PCE and t.

In the most efficient HPSCs with the best PV performance, PbI_2_ is the main Pb-containing decomposition product, and thus is likely the main product to leak from broken solar modules due to its easy water solubility. The U.S. Environmental Protection Agency (EPA) has identified lead as 1 of 15 pollutants often found in publicly owned treatment works (POTW) and sewage that it considers a potential pollutant of concern [46]. The PbI_2_ solubility in water is 0.76 g L^−1^ at 20 °C [47], while the maximum accepted levels of Pb in drinking water are set to be five orders of magnitude lower, at 0.000015 g L^−1^ (15 ppb), by the EPA [46]. These numbers manifest the importance of limiting the possible leaching of dissolved Pb-containing products from HPSCs into the environment. One strategy to mitigate Pb leaching from HPSCs into the environment is through the use of encapsulants. Also, encapsulation for solar panels is a critical issue for the long-term operational stability of HPSCs. In the second phase of this cost analysis, we evaluated the perovskite cost with four different cost-effective and commercially available polymer-based encapsulants viable for academic research, such as ethylene–vinyl acetate (EVA), Polyolefin (TPO), Polytetrafluoroethylene (PTFE) known by its trade name Teflon^®^, and Polyethylene terephthalate (PET).

Currently, the most common polymeric encapsulant material used in commercial silicon solar modules is EVA, due to its low-cost and easy processability [48,49]. It offers a low water vapor transmission rate (WVTR) compared to some other encapsulants reported in the literature but a high water diffusion rate, causing a possible decline in the module lifetime [50]. Also, its sensitivity to discoloration under UV radiation results in decreased light-transmittance, and therefore reduced solar cell power output [49]. Polyolefin is a commonly used encapsulant in academic research and has several advantages like a good adhesion energy, creep failure resistance, low WVTR, low discoloration rate, and better light transmittance compared to EVA [51,52,53].

Studies report the unique use of hydrophobic fluoropolymer, PTFE, for improving the perovskite crystallinity and passivating defects when using an optimum amount as an additive in the perovskite organic precursor [54]. Studies also report that the hydrophobic passivation of a PTFE precursor solution prevents PbI_2_ decomposition and improves moisture stability [55]. However, it is sensitive to electrophilic attack upon reaction with alkali metals when exposed to long hours of heat [56]. Several studies have used PET in a hybrid encapsulation framework in combination with transparent metal oxide films (e.g., Al_2_O_3_) or inorganic/organic multilayers and reported its compatibility for use on flexible substrates [57,58,59]; however, it is comparatively costlier than the previously mentioned encapsulants. The USD/m^2^ values of these encapsulants are summarized in Table S3. Recent improvements in perovskite stability through these encapsulants are summarized in Table S4. There are many other effective encapsulants being studied for stability improvement in perovskite. A comprehensive cost analysis with additional highly efficient encapsulants will be discussed in a future review paper.

**Figure 3:**
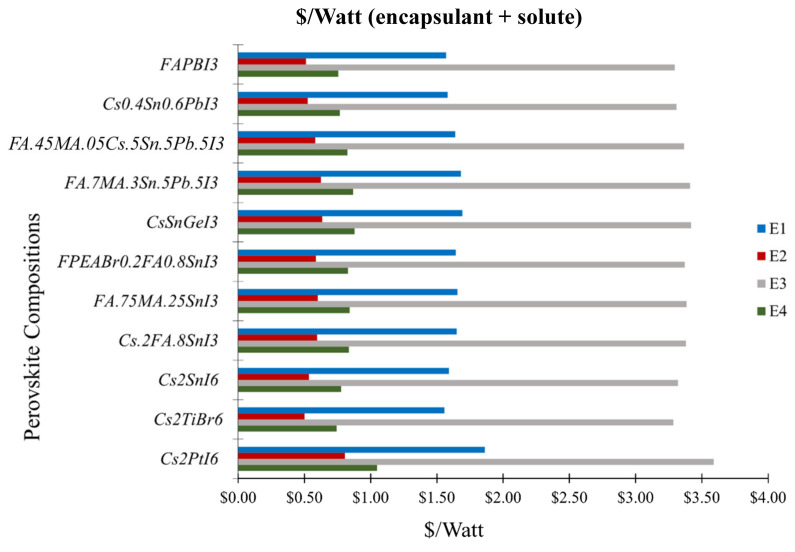
USD/Watt (solute + encapsulant) of various Pb and Pb-free perovskite compounds calculated with respect to the highest PCE of 25.6% and thickness of 2000 nm reported for the Pb-based FAPbI_3_ perovskite. E1, E2, E3, and E4 represent different encapsulants: Polyolefin, Teflon, PET, and EVA, respectively. Figure S2 represents the USD/Watt (solute + encapsulant) calculated with respect to the PCE and absorber layer thickness reported in the corresponding literature. Figure S3 represents the USD/watt (solute + encapsulant) with the discrete effect of optimized PCE reported for the Pb-based FAPbI_3_ perovskite and the corresponding absorber layer thickness from the literature. Figure S4 represents the USD/watt (solute + encapsulant) with the discrete effect of the optimized absorber layer thickness reported for the Pb-based FAPbI_3_ perovskite and PCE from the literature.

Figure 3 is the graphical representation of the USD per watt of solutes with the added encapsulant cost of several perovskite compositions, considering each perovskite composition with an optimized PCE of 25.6% and t of 2000 nm as reported for the state-of-art FAPbI_3_ perovskite. If both the parameters are optimized, the USD/watt of the (solute + encapsulant) Cs_2_PtI_6_ is estimated to be only ~1–1.4% more expensive than the FAPbI_3_ perovskite for four different encapsulants. Among them, the most expensive PET renders a cost of ~3.6 USD/watt for Cs_2_PtI_6_ (3.3 USD/watt for FAPbI_3_) and the least expensive Teflon renders a cost of ~0.81 USD/watt for Cs_2_PtI_6_ (0.516 USD/watt for FAPbI_3_), which are ~2.5-times and ~4.7-times cheaper than the USD/watt values of solutes+encapsulants with its reported PCE of 13.88% and t of 10,000 nm as outlined in Figure S2. The calculated precursor cost of FAPbI_3_ with expensive encapsulants like PET (3.29 USD/watt) exceeds that of the unencapsulated Cs_2_PtI_6_ (2.89 USD/watt). Figures S3 and S4 represent the USD/watt value (solute + encapsulant) with the discrete effect of the optimized PCE and optimized absorber layer thickness reported for the Pb-based FAPbI_3_ perovskite, respectively.

The long-term operational stability investigated using maximum power point (MPP) tracking under a simulated 1-sun illumination for the unencapsulated FAPbI_3_-based PSC reports a loss of 15% of its initial efficiency under constant light exposure using an LED lamp for 450 h at around 35 °C [2]. The unencapsulated Cs_2_PtI_6_-based device tested under AM1.5G at 65 °C for 500 h shows a loss of 23% of its initial efficiency. The shunts causing a decrease in Voc and FF after light-soaking are expected to develop due to pinholes in the Cs_2_PtI_6_ films and can be improved with film quality [15]. Considering the high water solubility of the Pb-based compound, a strong and effective encapsulant system is needed which can potentially increase the overall cost. With comparable efficiency and t, Cs_2_PtI_6_ can be considered a suitable alternative to Pb-based perovskites despite the high cost associated with Pt-based solutes. However, considering the high cost associated with its current PCE and t, Cs_2_PtI_6_ is not viable for commercialization, and exploration for alternative compounds to replace Pt has immense research significance. In this paper, we have summarized the primary results corresponding to the partial replacement of Pt with Ni in the B-site.

### 3.2. Pt-Ni Mixing in Halide Perovskite

**Figure 4:**
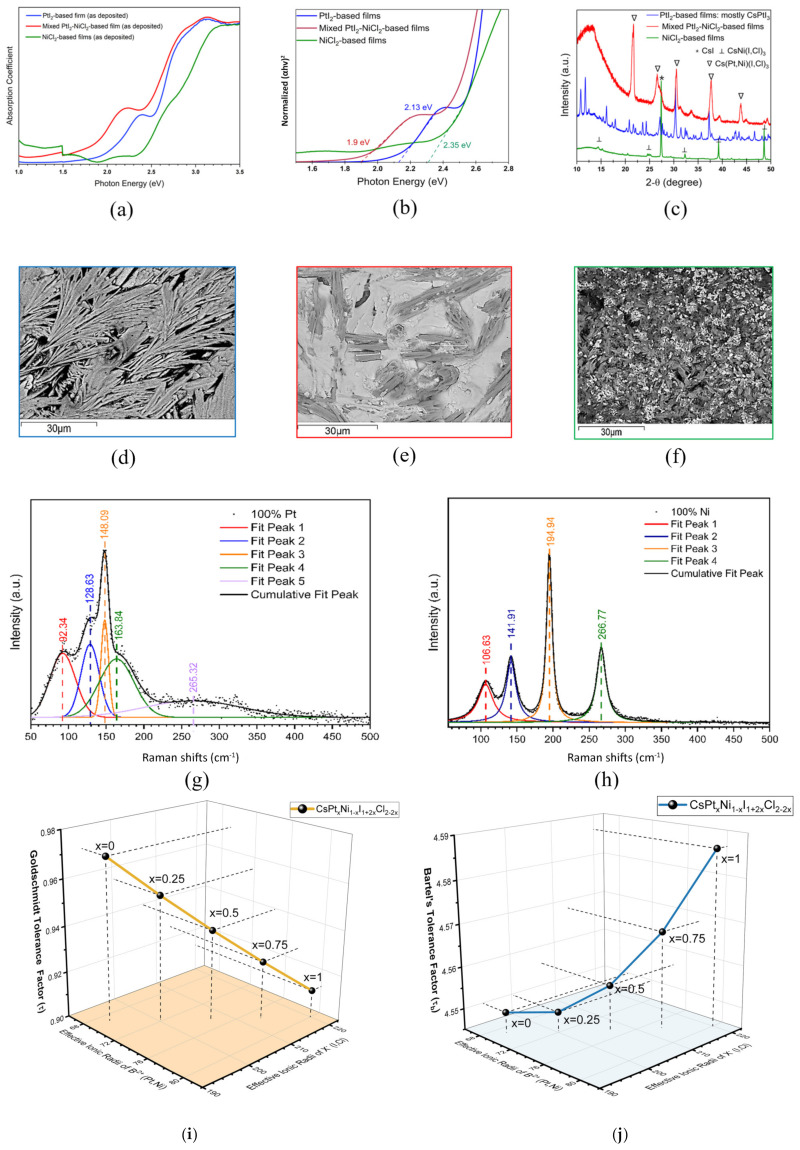
(**a**) Absorption spectrums of 2 h annealed (at −15 in Hg and 100 °C) PtI_2_, mixed PtI_2_-NiCl_2_, and NiCl_2_-based films; (**b**) Tauc plot showing the optical bandgap of the 2 h annealed (at −15 in Hg and 100 °C) PtI_2_, mixed PtI_2_-NiCl_2_, and NiCl_2_-based films; (**c**) XRD spectra of the 2 h annealed (at −15 in Hg and 100 °C) PtI_2_, mixed PtI_2_-NiCl_2_, and NiCl_2_-based films; SEM images of (**d**) PtI_2_, (**e**) mixed PtI_2_-NiCl_2_, and (**f**) NiCl_2_-based films; Raman spectra of (**g**) PtI_2_-based and (**h**) NiCl_2_-based films, respectively; (**i**) Goldschmidt and (**j**) Bartel tolerance factors for Cs(Pt,Ni)(Cl,I)_3_.

Figure 4a shows the absorption spectrum of the PtI_2_-based films, mixed PtI_2_-NiCl_2_-based films, and NiCl_2_-based films, respectively. To estimate the bandgap of these thin films, the Tauc plots shown in Figure 4b are derived from the absorption coefficient based on Equation (5):

(5) $${\left(\alpha hv\right)}^{n}=A\left(hv-E_{g}\right)$$

where α is the absorption coefficient, hv is the photon energy, $E_{g}$ is the optical band gap energy, A is a proportionality constant, and *n* = 2 for direct allowed transition.

The bandgap analysis (Tauc plots) of our PtI_2_-based films, mixed PtI_2_-NiCl_2_-based films, and NiCl_2_-based films shows a bandgap of 2.13 eV for PtI_2_-based films. The bandgap increases to 2.35 eV when NiCl_2_ entirely replaces PtI_2_. Alteration of the halide anion changes the bond distance and/or angle of X–B–X, and the incorporation of a smaller X anion, such as Cl replacing I, increases the bandgap [60]. Our bandgap analysis of NiCl_2_-based films replacing PtI_2_ supports this theory. The partial substitution of NiCl_2_ with PtI_2_ in mixed PtI_2_-NiCl_2_-based films is supposed to exhibit a larger bandgap than that of the NiCl_2_-based films upon the incorporation of the larger Pt cation in the B-site. However, the reduced bandgap of 1.9 eV in mixed PtI_2_-NiCl_2_-based films is likely driven by the incorporation of the larger X anion (I- in this case) rather than the larger B cation.

The XRD analysis of the three types of films is shown in Figure 4c. The XRD pattern shows mixed phases of many unidentified lower-intensity diffraction peaks with a partial match to Cs_2_PtI_6_ resulting in a higher relative noise level in the films prepared in the CsI + PtI_2_ precursor and indicates a poor crystallinity. A peak shift to higher angles in XRD can be attributed to the incorporation of B-site cations with larger ionic radii, leading to an expansion of the perovskite lattice [61,62,63]. Similar results are observed in our study with the inclusion of the larger Pt atoms in the mixed PtI_2_-NiCl_2_-based films. The formation of the Cs(Pt,Ni)(I,Cl)_3_ phase in the mixed PtI_2_-NiCl_2_-based films is confirmed by a peak shift of standard CsNiCl_3_ (a = 7.118 Å, b = 7.118 Å, c = 5.9085 Å) to a higher 2-theta. The diffraction peaks of this new phase are located in between the CsNiCl_3_ and Cs_2_PtI_6_ phases, which implies the intercalation of Pt into the perovskite lattice and the formation of a mixed Pt-Ni-based phase (Table 1).

In the XRD spectra, a new phase of CsNi(I,Cl)_3_ is formed with the complete substitution of Pt with Ni, as evidenced by a shift of the XRD peak to a lower 2θ compared to the standard XRD peak of CsNiCl_3_. The peaks of CsNiCl_3_ located at 20.8°, 25°, and 32.74° shift to 20.51°, 24.59°, and 32.3°, respectively, and form this mixed anion phase in the CsI + NiCl_2_ precursor. The peak located at 27.4° in the XRD pattern of the NiCl_2_-based films was identified as CsI (27.7°) with a peak shift to a lower angle (27.39°). The wide bandgap in NiCl_2_-based films may also be ascribed to the presence of CsI.

SEM images of the three types of films are presented in Figure 4d–f. PtI_2_-based films have a long needle-like structure, which is completely different than our previous observation in films prepared in the CsI + PtI_4_ precursor. This SEM morphology along with the XRD pattern and bandgap provides strong evidence that we are not making a pure phase of Cs_2_PtI_6_ with the CsI + PtI_2_ precursor [15].

Raman spectroscopy measurements were also conducted on the NiCl_2_-based films and PtI_2_-based film, as shown in Figure 4g and Figure 4h, respectively. The sharp Raman peaks detected on the NiCl_2_-based film around 107, 142, 195, and 267 cm^−1^ are in line with reported values from the literature [66,67,68]. The peak at 267 cm^−1^ matched well with the A1g modes of CsNiCl_3_; the peak near 195 cm^−1^ correlated with the E_2g_ mode, while the peak at 142 cm^−1^ was attributed to the E_1g_ modes of CsNiCl_3_ [66]. As the film system also contained I- (with the introduction of CsI), the unassigned peak at ~106.63 might come from a Ni-I or CsNiI_3_ structure [69]. The Raman peaks on the PtI_2_-based film were also observed and fitted at 92, 129, 148, 164, and 265 cm^−1^, respectively. These peaks are also in line with reported values from the literature [70,71,72]. The peak at ~129 and ~148 cm^−1^ should be assigned to the symmetric Pt-I stretch in ν2 mode (Eg) and ν1 mode (A_1g_), respectively [70,72]. And Raman shift peaks at ~93 and 160 cm^−1^ were also reported in Hexaiododiplatinate (II) salts, A_2_Pt_2_I_6_ [70]. This structure consists of Pt_2_I_6_ units and the corresponding cation (Cs in our case), while the anions form edge-shared squares [73]. The peak at ~93 cm^−1^ might come from the asymmetric I-Pt-I bend in ν4 mode (T1u) [72].

In order to elucidate the phase formation in PtI_2_-based films, we performed EDS analysis showing the average percentages of elements in the thin films, as shown in Figure S5. It indicates a phase with Cs:Pt:I in the intended precursor ratio of 1:1:3. However, no standard XRD pattern is available for CsPtI_3_ in the database, so our XRD analysis could not confirm the presence of this phase. Figure 4e shows two different microstructures, a dark rod-like structure and a transparent plate-like structure, present in mixed PtI_2_-NiCl_2_-based films. Similarly, NiCl_2_-based films have a dark rod-like microstructure with white particles on the film surface as presented in Figure 4f. The EDS analysis performed in various spectra of these films, as shown in Figures S6 and S7, indicates that the bright white plate-like features (solid columns) have a Cs-I-rich morphology, and the dark rod-like features (patterned columns) have a Cl-Ni-rich morphology, which suggests that the insolubility of NiCl_2_ is responsible for the formation of the rod-like features. In our previous study on mixed Sn-Pb perovskite [45], a similar microstructure was observed as a result of the coagulation tendency of the Pb compound due to the insolubility of PbI_2_. The presence of excess Cs and I particles observed in EDS analysis further validates the presence of CsI as identified in the XRD spectra of the NiCl_2_-based films. The EDS analysis also suggests the presence of a higher at% of sulfur in the rod-like surfaces rather than in the white Cs-I-rich surfaces, both in the mixed PtI_2_-NiCl_2_-based and NiCl_2_-based films. Even NiCl_2_-based films have almost no sulfur present in their Cs-I-rich regions. However, the influence of sulfur in the formation of rod-like features is not yet clear.

Moreover, we calculated the Goldshmidt and Bartel tolerance factor for the Cs(Ni,Pt)(I,Cl)_3_ films, as shown in Figure 4i and Figure 4j, respectively. The Goldshmidt tolerance factor and Bartel tolerance factor are shown in Equations (6) and (7), respectively.

(6) $$t=\frac{r_{A}+r_{X}}{\sqrt{2}\left(r_{B}+r_{X}\right)}$$

where $r_{A}$, $r_{B}$, and $r_{X}$ are the effective ionic radii for the ions A, B, and X.

(7) $$\tau =\frac{r_{x}}{r_{B}}-n_{A}(n_{A}-\frac{\frac{r_{A}}{r_{B}}}{ln\frac{r_{A}}{r_{B}}})$$

where “*n*” is the oxidation state; “*r*” denotes the ionic radius; and the subscripts “*A*”, “*B*”, or “*X*” denote the cation or anion of the ABX_3_ or A_2_BX_6_ structure.

It should be noted that the optimal stability range of the 3D perovskite structure is indicated in the window 0.8 < t < 0.9 for the Goldshmidt factor [74], while a Bartel’s tolerance factor < 4.18 predicts stable perovskite phase [75]. The tolerance factors of the Cs(Ni,Pt)(I,Cl)_3_ films are outside the optical range, which might explain the phase separation we observed.

**Figure 5:**
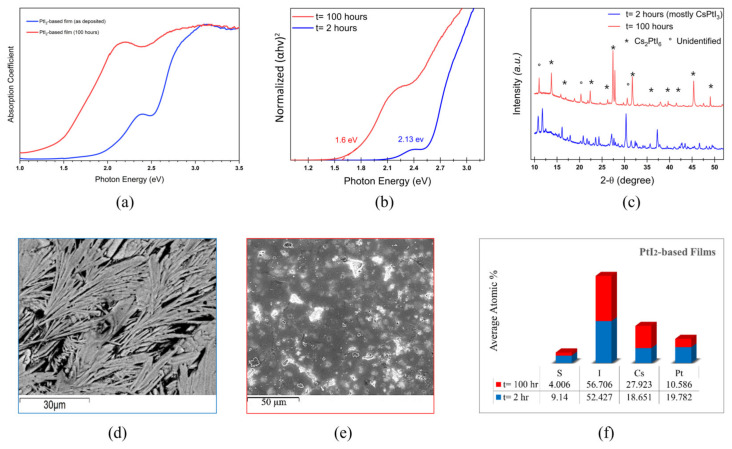
PtI_2_-based films before and after the dark thermal annealing test with t representing the annealing duration: (**a**) absorption coefficient; (**b**) Tauc plot; (**c**) XRD pattern; (**d**) cross-section SEM images before annealing; (**e**) cross-section SEM images after annealing; and (**f**) EDS analysis showing the atomic % of the elemental distribution.

In order to demonstrate the effect of long-time thermal annealing, we performed a heat-stability test by annealing the films for 100 h at 65 °C and compared the optoelectronic features to those of the reference films that were annealed for 2 h. Figure 5 indicates that the wide bandgap intermediate phase transforms to a stable Cs_2_PtI_6_ perovskite phase after long-time thermal annealing. Figure 5a shows the absorption spectrum of the PtI_2_-based films before and after the 100 h long thermal annealing. The absorption edge significantly red-shifted to a lower energy. The derived Tauc plots from the absorption coefficient in Figure 5b exhibit a significantly reduced bandgap of 1.6 eV in the PtI_2_-based films exposed to long thermal annealing, which is in reasonable agreement with the bandgap of the Cs_2_PtI_6_ film (1.4 eV) reported in our previous work [15]. It is known that crystallinity is critical for perovskite stability because the main defect-induced degradation starts near the grain boundaries [2]. The poor crystallinity in the reference PtI_2_-based films is consistent with poor optical measurements, which is improved with long-time thermal annealing. Upon thermal annealing, the PtI_2_-based films become more crystalline with a better match to the diffraction pattern of the Cs_2_PtI_6_ perovskite (11.361 Å) with some unreacted Cs residue (Figure 5c). The Cs_2_PtI_6_ phase was determined by the (111), (200), (220), (222), (400), (440), and (622) peaks. The amorphous morphology becomes compact including some Cs-rich white particles on top as confirmed by the SEM analysis in Figure 5d,e. The EDS analysis in Figure 5f further validates the formation of Cs_2_PtI_6_ in the thermally annealed films, showing a phase of Cs:Pt:I at an atomic ratio of 2:1:6 with some excess Cs, which is well aligned with our XRD analysis.

**Figure 6:**
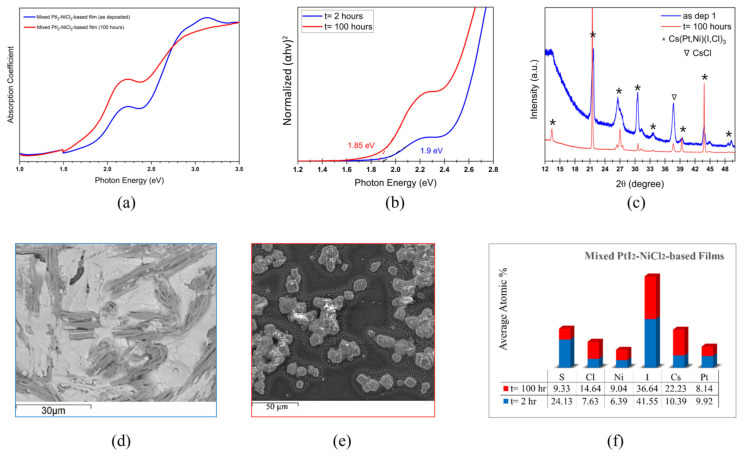
Mixed PtI_2_-NiCl_2_-based films before and after the dark thermal annealing test with t representing the annealing duration: (**a**) absorption spectrum; (**b**) Tauc plot; (**c**) XRD pattern; (**d**) cross-section SEM image before annealing; (**e**) cross-section SEM image after annealing; and (**f**) EDS analysis showing the atomic % of the elemental distribution.

The effect of thermal annealing on the optoelectronic properties of mixed PtI_2_-NiCl_2_-based films is demonstrated in Figure 6. The absorption spectra of the mixed PtI_2_-NiCl_2_-based films before and after thermal annealing is shown in Figure 6a. The absorption edge slightly red-shifted after thermal annealing. Figure 6b shows red-shifted bandgap spectra rendering a bandgap of 1.85 eV, very close to the bandgap (1.9 eV) of the reference films annealed for 2 h, possibly implying the stability of the mixed thin film against thermal annealing.

The peak broadening of the XRD pattern is inversely correlated with the crystallite size. Perovskite films with large crystallite sizes can have reduced grain boundaries and restrained carrier recombination, which increases carrier mobility [76]. Films exposed to extended thermal annealing have improved crystallinity, and narrow refined XRD spectra with a shift to higher angles as shown in Figure 6c, which can be correlated with reduced lattice parameters and is similar to our previous observation [45]. Similar to the reference films, thermally exposed films exhibit diffraction patterns in the same orientation of Cs(Pt,Ni)(I,Cl)_3_. The peak located at 37.56° in both of these films is attributed to the CsCl phase, which can be ascribed to the wider secondary bandgap. No significant change in the XRD pattern is observed, supporting our proposition of its thermal stability speculated through bandgap analysis. According to Figure 6d,e, the rod-like morphology transformed into a compact plate-like structure displaying white Cs-rich crystals on the surface.

According to the EDS analysis in Figure 6f, a significant decline in the atomic distribution of sulfur content is observed after the thermal treatment. We attribute this to the evaporation of the DMSO solvent residue upon annealing which might be responsible for the refined and crystalline XRD spectra.

**Figure 7:**
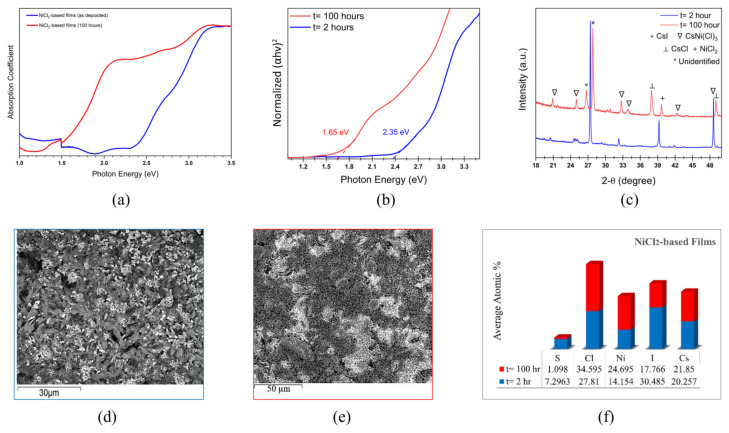
NiCl_2_-based films before and after the dark thermal annealing test with t representing the annealing duration: (**a**) absorption spectrum; (**b**) Tauc plot; (**c**) XRD pattern; (**d**) SEM morphology before annealing; (**e**) SEM morphology after annealing; and (**f**) EDS analysis showing the atomic % of the elemental distribution.

The effect of thermal annealing on the optoelectronic properties of NiCl_2_-based films is demonstrated in Figure 7. The absorption spectrum of the NiCl_2_-based films before and after the thermal treatment is shown in Figure 7a. The absorption edge shifted from 500 nm to 750 nm, confirming the suppressed bandgap achieved in the thermally exposed films. The Tauc plot shown in Figure 7b exhibits a significantly narrower bandgap of 1.68 eV in the films annealed for 100 h, compared to the films annealed for 2 h (2.35 eV). These films have improved crystallinity and better match CsNiCl_3_, confirmed by a shift of CsNi(I,Cl)_3_ to higher angles as shown in Figure 7c. Similar to the mixed PtI_2_-NiCl_2_-based films, the peak located at 37.56° is attributed to the CsCl phase.

According to Figure 7d,e, the rod-like features in the reference films transform into a more plate-like compact morphology.

The EDS analysis in Figure 7f confirms the suppression of I- upon thermal treatment, suggesting an agreement with the suppressed CsI as detected in the XRD analysis. The reduction in the CsI intensity may be correlated with the reduced bandgap achieved after 100 h of thermal annealing. Similar to PtI_2_-based films and mixed PtI_2_-NiCl_2_-based films, a decline in the sulfur content was also observed in the thermally annealed NiCl_2_-based films.

Several research groups have studied the excellent performance of Ni-based compounds (e.g., NiI_2_) in ultra-low humidity, which is attributed to their susceptibility to humidity and the resulting material transition characteristics. Zhang et al. [34] studied the moisture-induced discoloration of NiI_2_ in humidity detection. Their study suggests that this moisture-induced material transformation of NiI_2_ leads to a change in the bulk resistance of the materials due to a change in material composition, indicating that the crystal structure of NiI_2_ is not directly affected by temperature. Rather, temperature promotes the rapid desorption of water molecules from the materials. Their conclusion also suggests that the hydrophilicity of this compound is reversible, meaning the transition between NiI_2_ and NiI_2_•6H_2_O due to the absorption of water in the presence of wet-environment molecules and the desorption of water by removing them is reversible. Studies have also confirmed that the dark state of the film (in a dry environment) leads to higher absorbance compared to the transparent state (in a wet environment) [34,77]. We also observed a transparent state of the NiCl_2_-based films before thermal annealing and a reversible color change (black ↔ orange) after 2 h of thermal annealing in a vacuum chamber, as depicted in Figure S8. The films exhibit a dark state for a remarkably long period after 100 h of thermal annealing, which is attributed to the greater stability achieved through thermal annealing. The lighter state of the film exhibits close to 40–60% optical transmittance between 350 and 1400 nm, while the dark states exhibit less than 20% optical transmittance through the visible spectrum, with a gradually increasing transmittance through the near-IR spectrum (Figure S9). This observation in our study is well positioned with respect to previous studies [34,77]. However, in the case of the mixed PtI_2_-NiCl_2_-based composition, we observed neither the transparent state nor a rapid color transformation, which triggered us to explore the optical properties of the films upon NiCl_2_ inclusion.

## 4. Conclusions

In this study, we first analyze the precursor materials’ cost for state-of-art Pb and Pb-free halide perovskites and encapsulation materials. Cs_2_PtI_6_ shows comparable optoelectronic properties to the highly efficient FAPbI_3_; however, due to the high cost of PtI_4_ and the reported absorber layer thickness, the precursor cost is estimated to be 15-times higher. Commercially available encapsulants such as Teflon, PET, EVA, and polyolefin have been used for the cost analysis. We find a trade-off between the encapsulant-enhanced stability of FAPbI_3_ and the cost of Pt in air-stable Cs_2_PtI_6_. The precursor cost of unencapsulated Cs_2_PtI_6_ (2.89 USD/watt) is estimated to be cheaper than encapsulated FAPbI_3_ with expensive encapsulants like PET (3.29 USD/watt); and with comparable efficiency and absorber layer thickness, PET-encapsulated Cs_2_PtI_6_ (3.59 USD/watt) is expected to cost just 1.1-times more. Considering the high water solubility and toxicity of Pb-based perovskites, expensive encapsulants are needed for the commercialization of FAPbI_3_ solar cells. To evaluate the replacement of Pt in Cs_2_PtI_6_, we also explore the substitution of Pt with Ni in the second part of this study. The structure, bandgap, and stability of Cs(Pt_x_Ni_1−x_)(I,Cl)_3_ thin films are evaluated for x = 1, 0.5 and 0. We synthesize perovskite films using the doctor-blade method with CsI, PtI_2_, and NiCl_2_ in a 50%–50% DMF–DMSO solvent mixture. The precursor concentration (for Pt + Ni) is fixed at 0.25 M. The precursor mixture was heated at 75 °C for 1.5 h, followed by drop-casting on the preheated Tec10 substrate. The doctor-blade coating technique was used to spread the solution over the preheated substrates. Films were then annealed in a vacuum oven at −15 in Hg and 100 °C for 2 h. For stability testing, the films were exposed to a dark thermal annealing test at 65 °C for 100 h. The PtI_2_-based films result in a bandgap of 2.13 eV and the XRD pattern shows an unidentified mixed amorphous phase with possible matches to CsPtI_3_ or Cs_2_PtI_6_. EDS analysis confirms PtI_2_-based films have a Cs:Pt:I atomic ratio of 1:1:3; however, due to lack of standard XRD patterns, the CsPtI_3_ phase is not confirmed. After thermal annealing for 100 h, PtI_2_-based films form crystalline structures that more closely match Cs_2_PtI_6_, as revealed by the XRD pattern and confirmed by the average atomic ratios obtained from EDS measurements. With thermal annealing, the bandgap of the film reduces from 2.13 eV to 1.6 eV, with the latter being a closer match to the previously reported Cs_2_PtI_6_ phase. Films deposited with CsI + NiCl_2_ precursors result in a bandgap of 2.35 eV which is between the reported values of 0.8 eV for CsNiCl_3_ and 3.86 eV for CsI. SEM shows a mixed morphology of two phases: a rod-like structure identified as CsNi(I,Cl)_3_ and white particles of CsI, also confirmed by XRD. After annealing for 100 h, the bandgap of NiCl_2_-based films reduces to 1.65 eV, and XRD primarily shows the CsNiCl_3_ phase. This observation is also confirmed by the change in the appearance of the films from translucent in the as-deposited state to dark brown after annealing. With a 50-50 mixture of PtI_2_ and NiCl_2_, the resulting bandgap of 1.9 eV with the XRD pattern showing a close match to CsNiCl_3_ with a shift to a higher 2-theta confirms the substitution of Pt into the CsNiCl_3_ lattice. With thermal annealing, the films show improved crystallinity, and the bandgap is stable at 1.85–1.9 eV. Our study shows the promise of creating earth-abundant halide perovskites such as CsNiCl_3_ or Cs(Pt,Ni)(I,Cl)_3_ to address the stability and toxicity issues of FAPbI_3_. Future work should include studies of the charge transport and other optoelectronic properties of Cs(Pt,Ni)(I,Cl)_3_, which shows the best stability in our study.

## Figures and Tables

**Table 1:** The 2-theta values of the mixed PtI_2_-NiCl_2_-based phase.

Structure	2Ɵ	2Ɵ	2Ɵ	Ref.
Std. CsNiCl_3_ (ICSD: 423828)	20.8°	25°	30.24°	[64]
New Structure: Cs(Pt,Ni)(I,Cl)_3_	21.68°	26.56°	30.52°	Our work
Std. Cs_2_PtI_6_ (ICSD: 37193)	22.52°	27.8°	32.14°	[65]

## Data Availability

The original contributions presented in this study are included in the article/Supplementary Material. Further inquiries can be directed to the corresponding author.

## Supplementary Materials

The following supporting information can be downloaded at https://www.mdpi.com/article/10.3390/ma17246196/s1, Figure S1: Discrete effect of optimized PCE and absorber layer thickness in perovskite cost analysis. $/Watt (solute) of various Lead and Lead-free perovskite compositions; Figure S2: $/Watt (solute+ encapsulant) of various Lead and Lead-free perovskite compounds calculated with respect to the PCE and absorber layer thickness reported in the corresponding literature. E1, E2, E3, and E4 represent different encapsulants, such as Polyolefin, Teflon, PET, and EVA, respectively; Figure S3: Effect of PCE in perovskite cost analysis: $/Watt (solute + encapsulant) of various Lead and Lead-free perovskite compositions calculated with respect to the highest PCE of 25.6% reported for the Lead-based FAPbI_3_ perovskite and absorber layer thickness reported in the corresponding literature. E1, E2, E3, and E4 represent different encapsulants, such as Polyolefin, Teflon, PET, and EVA, respectively; Figure S4: Effect of absorber layer thickness in perovskite cost analysis: $/Watt (solute + encapsulant) of various Lead and Lead-free perovskite compounds calculated with respect to the PCE reported in the corresponding literature and absorber layer thickness of 2000 nm reported for the Lead-based FAPbI_3_ perovskite. E1, E2, E3, and E4 represent different encapsulants, such as Polyolefin, Teflon, PET, and EVA, respectively; Figure S5: EDS analysis of average at.% of elemental distribution in PtI_2_-based films featuring a microstructure of Cs:Pt:I= 1:1:3; Figure S6: EDS analysis of average at.% of elemental distribution in mixed PtI_2_-NiCl_2_-based films featuring different microstructures present in the film surface; Figure S7: EDS analysis of average at.% of elemental distribution in NiCl_2_-based films featuring different microstructures present in the film surface; Figure S8: Moisture-induced discoloration in NiCl_2_-based films before and after the dark thermal anneal test; Figure S9: Temperature-dependent transmittance in NiCl_2_-based films; Table S1: Published Molecular Mass and Cost per gm of Each Solute; Table S2: PCE and Absorber Layer Thickness of Pb and Pb-free Perovskites Reported in Literatures; Table S3: Encapsulation Cost for Perovskite Stability; Table S4: Summary of Recent Improvements in Perovskite Stability through Different Encapsulants.
