# Supplementary material for: Mixed Pt-Ni Halide Perovskites for Photovoltaic Application
Source: Materials (Basel). 2024 Dec 18;17(24):6196. doi: 10.3390/ma17246196 (PMC11677164; doi:10.3390/ma17246196)
Supplement: Supplementary file 1 [file materials-17-06196-s001.zip › materials-3341003-supplementary.pdf]

Table S1. Published Molecular Mass and Cost per gm of Each Solute

Compound	Molecular Weight (gm/mol)	Weight (g)	Price ($)	S/gm	Purity	CAS
CsI	259.81	100	236	2.36	99.90%	7789-17-5
FAI	171.97	25	387	15.48	98%	879643-71-7
MAI	158.97	25	622	24.88		14965-49-2
SnI_2	372.52	5	165	33	99.99%	10294-70-9
PbI_2	461.01	50	58.8	1.176		10101-63-0
GeI_2	326.45	1	184	184	99.80%	13573-08-5
$TiBr_4$	367.48	100	312	3.12	98%	7789-68-6
PtI_4	702.7	1	226.55	226.55	99	Alpha Aesar
FPEABr	203.05	5	73.5	14.7	97	332-42-3
CsBr	212.81	100	230	2.3	99.90%	7787-69-1

Table S2. PCE and Absorber Layer Thickness of Pb and Pb-free Perovskites Reported in Literatures.

#	Compositions	Molarity (M)	Thickness (nm)	Reported PCE(%)	Ref.
**1**	Cs_2PtI_6	0.25	10,000	13.88	[1]
**2**	Cs_2TiBr_6	0.5	200	3.3	[2]
**3**	Cs_2SnI_6	0.4	2000	5.18	
**4**	$Cs_{0.2}FA_{0.8}SnI_3$	0.9	250	10.8	[3]
**5**	$FA_{0.75}MA_{0.25}SnI_3$	0.9	190	11.5	[4]
**6**	$FPEABr_{0.2}FA_{0.8}SnI_3$	0.8	200	14.81	[5]
**7**	$CsSnGeI_3$	0.5	200	7.11	[6]
**8**	$FA_{0.7}MA_{0.3}Sn_{0.5}Pb_{0.5}I_3$	1.8	1000	20.74	[7]
**9**	$FA_{0.5}MA_{0.45}Cs_{.05}Sn_{0.5}Pb_{.5}\ I_3$	1.2	1000	20.2	[8]
**10**	$Cs_{0.4}Sn_{0.6}PbI_3$	0.7	500	13.37	[9]
**11**	$FAPBI_3$	0.8	2000	25.6	[10]

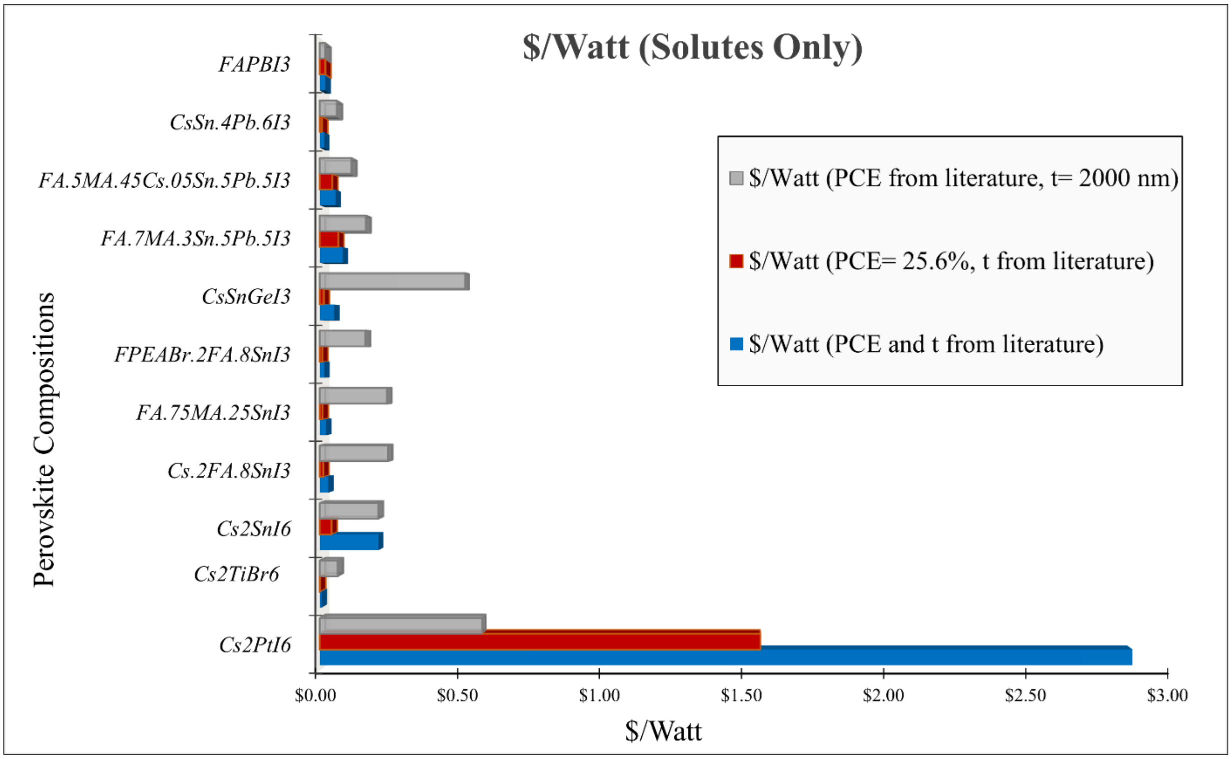

Figure S1. Discrete effect of optimized PCE and absorber layer thickness in perovskite cost analysis. $/Watt (solute) of various Lead and Lead-free perovskite compositions

Table S3. Encapsulation Cost for Perovskite Stability.

#	**Encapsulants**	**Description**	**Specification**	**Area (m^2)**	**Quantity**	**$/pkg**	**$/film**	**$/m2**	**Source**
**E1**	Polyolefin film	non-sterile, universal optical sealing	7.8 cm × 14.1 cm	0.011	100	436	4.36	396.4357	CLS6575-100EA
**E2**	Teflon	non-sterile	W= 1 in, roll size=520 in	0.3355	1	42.3	42.3	126.0805	Z221880-1PAK
**E3**	PET	polyester, sheet, condition amorphous	t= 1mm, 600mm*600mm	0.36	1	302	302	838.8889	GF59152350-1EA
**E4**	EVA	Eva-4 Foam Sheet, Water-Resistant Closed Cell	12 in*12 in	0.093	1	17.5	17.5	$188.17	eBay

Table S4. Summary of Recent Improvements in Perovskite Stability through Different Encapsulants

HPSCs Stack	Encapsulant	Stability	Ref.
ITO/NiO$_x$/Cs$_{0.1}$FA$_{0.7}$MA$_{0.2}$PbI$_x$Br$_{3-x}$/PC$_{61}$BM/BCP/Ag	EVA	Retained more than 95% of the initial PCE after 1000 h at inert conditions (≈40% RH)	11
FTO/TiO$_2$/MAPbI$_3$/ spiro-MeOTAD/Ag	EVA	Stable after 350 h at UV irradiation	12
ITO/Cs$_{0.17}$FA$_{0.83}$Pb(Br$_{0.17}$I$_{0.83}$)$_3$/LiF/PC$_{60}$BM/SnO$_2$/ Sputtered ITO	EVA	Retained over 90% of its initial PCE after 200 temperature cycles	13
glass/ITO/NiO/Cs$_{0.17}$FA$_{0.83}$Pb(Br$_{0.17}$I$_{0.83}$)$_3$/LiF/ PCBM/SnO$_2$/ZTO/ITO/Ag	EVA cover adhesive + butyl rubber edge sealant	No significant PCE decline after 1000 h damp test and 200 cycles of temperature cycling test (IEC 61215)	14
PET/EVA/ITO-PET/ NiOx/MAPbI3/EVA/ PCBM/PEI/ITO-PET/EVA/PET	Eva-coated PET substrate	* PCE drops by 8% after 10000 hr storage test at 80% RH.<br>** Retains 85% of PCE after 30 crumpling cycles in water.	15
ITO/NiO$_x$/Cs$_{0.17}$FA$_{0.83}$Pb (Br$_{0.17}$I$_{0.83}$)$_3$/LiF/PC$_{60}$BM/SnO$_2$/ITO	Polyolefin	Stable after 1000 h of damp heat testing and	16

		1000 h of dry heat testing	
glass/ITO/NiO/$Cs_{0.17}FA_{0.83}Pb(Br_{0.17}I_{0.83})_3$*/LiF/PCBM/*$SnO_2$*/ITO/Ag*	polyolefin 'ENLIGHT' + butyl rubber edge seal	Negligible PCE decline after 1000 h dry (25% RH) and damp (85% RH) heat tests at 85 ◦C (IEC 61215)	[17]
FTO/c-TiO_2*/m-*TiO_2*/*$(FAPbI_3)_{0.85}(MAPbBr_3)_{0.15}$*/spiro-OMeTAD/Au*	Norland Optical Adhesive (NOA)/PET	Stable after 540 h at 25 °C	[18]
glass/ITO/PEDOT:PSS/$MAPbI_3$*/ALD-ZnO/Ag*	Al_2O_3-coated PET	Retained the initial PCEs after 1000 h at 30 ◦C and 65% RH	[19]
FTO/c-TiO_2*/m-*TiO_2*/*$MAPbI_3$*/spiro-OMeTAD/Au*	PTFE/Teflon	Retained 95% of its initial PCE after 30 days at ambient atmosphere	[20]
glass/FTO/c-TiO_2*/mp-*TiO_2*/*$MAPbI_3$*/spiro-OMeTAD/Au*	PTFE/Teflon	Initial PCE fully retained after immersion in water for 60 s 5% reduction of the initial PCE after 720 h at 25 ◦C, 50% RH, in dark	[20]

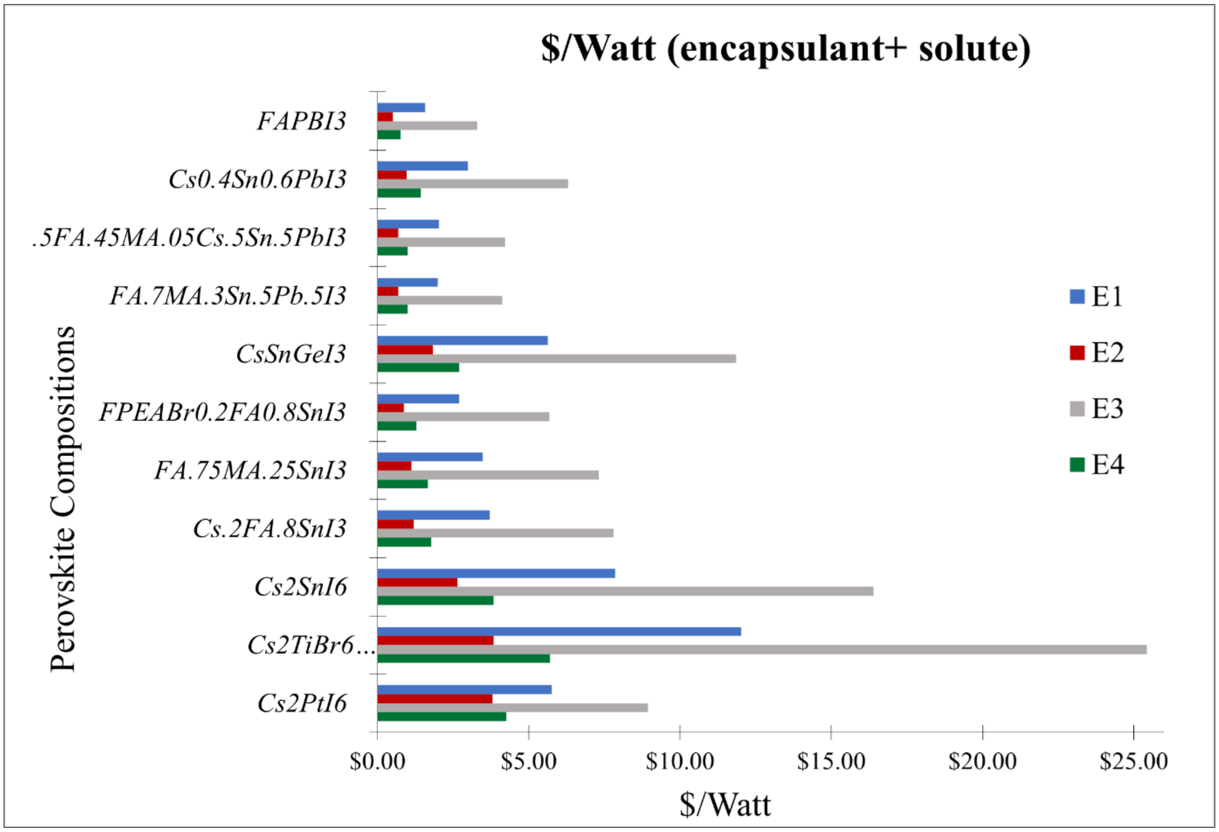

Figure S2. $/Watt (solute+ encapsulant) of various Lead and Lead-free perovskite compounds calculated with respect to the PCE and absorber layer thickness reported in the corresponding literature. E1, E2, E3, and E4 represent different encapsulants, such as Polyolefin, Teflon, PET, and EVA, respectively.

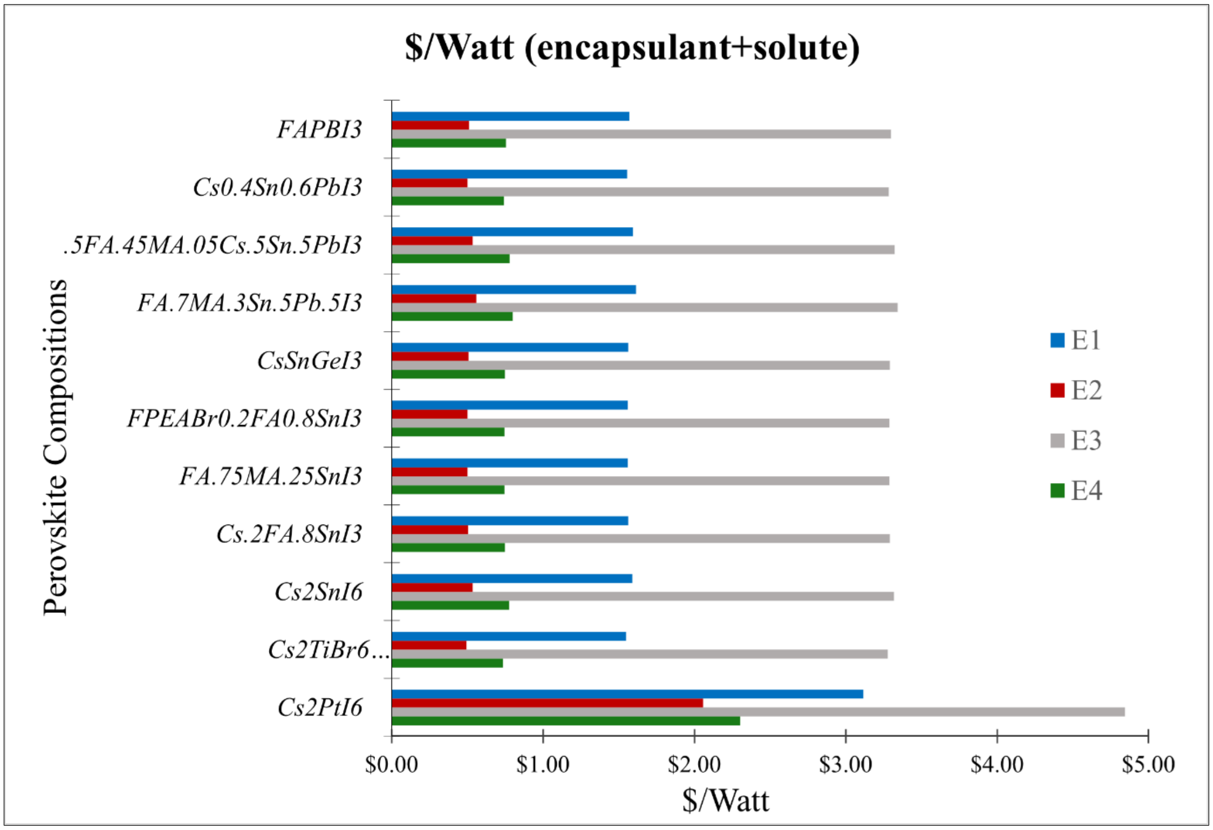

Figure S3. Effect of PCE in perovskite cost analysis: $/Watt (solute+ encapsulant) of various Lead and Lead-free perovskite compositions calculated with respect to the highest PCE of 25.6% reported for the Lead-based $FAPbI_3$ perovskite and absorber layer thickness reported in the corresponding literature. E1, E2, E3, and E4 represent different encapsulants, such as Polyolefin, Teflon, PET, and EVA, respectively.

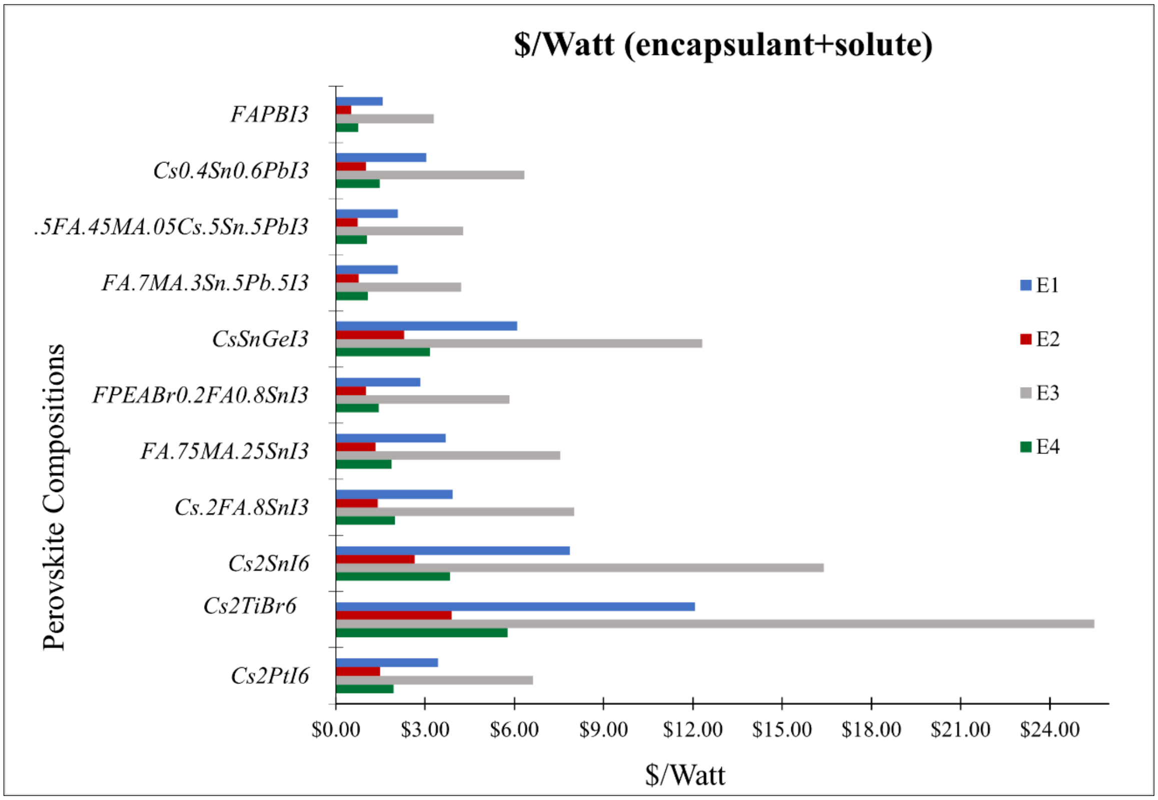

Figure S4. Effect of absorber layer thickness in perovskite cost analysis: $/Watt (solute + encapsulant) of various Lead and Lead-free perovskite compounds calculated with respect to the PCE reported in the corresponding literature and absorber layer thickness of 2000 nm reported for the Lead-based $FAPbI_3$ perovskite. E1, E2, E3, and E4 represent different encapsulants, such as Polyolefin, Teflon, PET, and EVA, respectively.

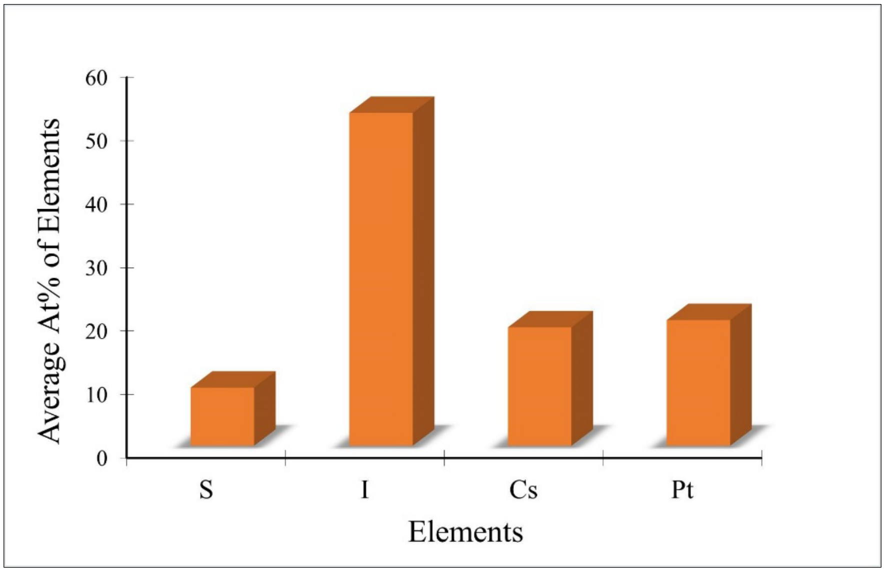

Figure S5. EDS analysis of average at.% of elemental distribution in PtI_2-based films featuring a microstructure of Cs:Pt:I= 1:1:3.

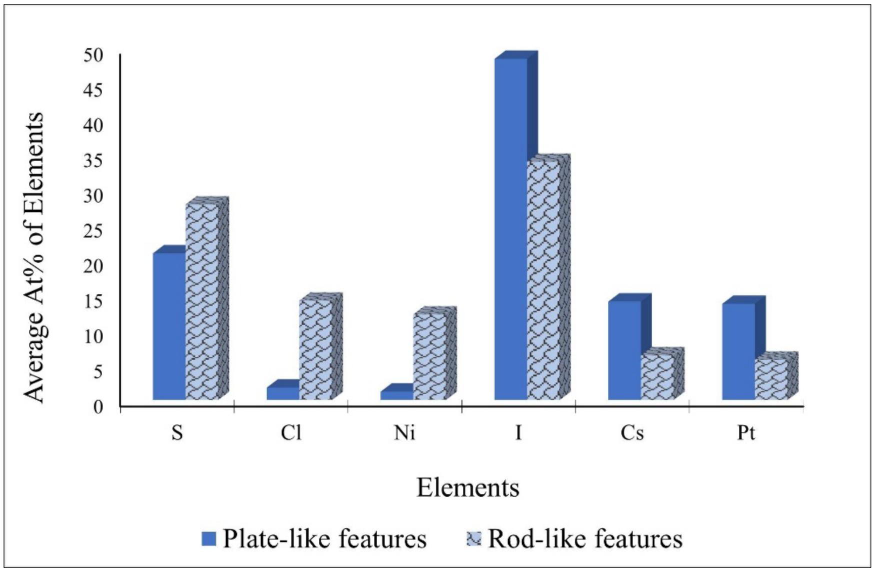

Figure S6. EDS analysis of average at.% of elemental distribution in mixed PtI_2-$NiCl_2$-based films featuring different microstructures present in the film surface.

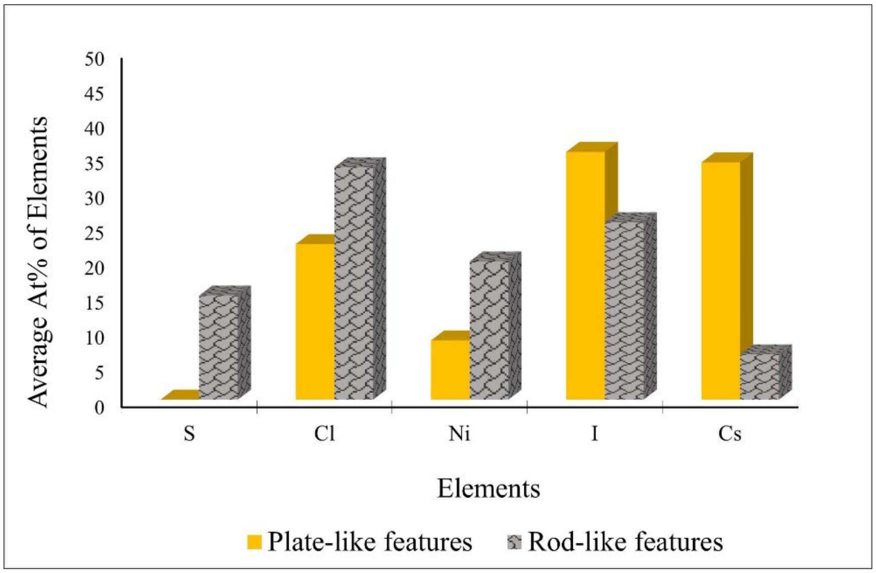

Figure S7. EDS analysis of average at.% of elemental distribution in $NiCl_2$-based films featuring different microstructures present in the film surface.

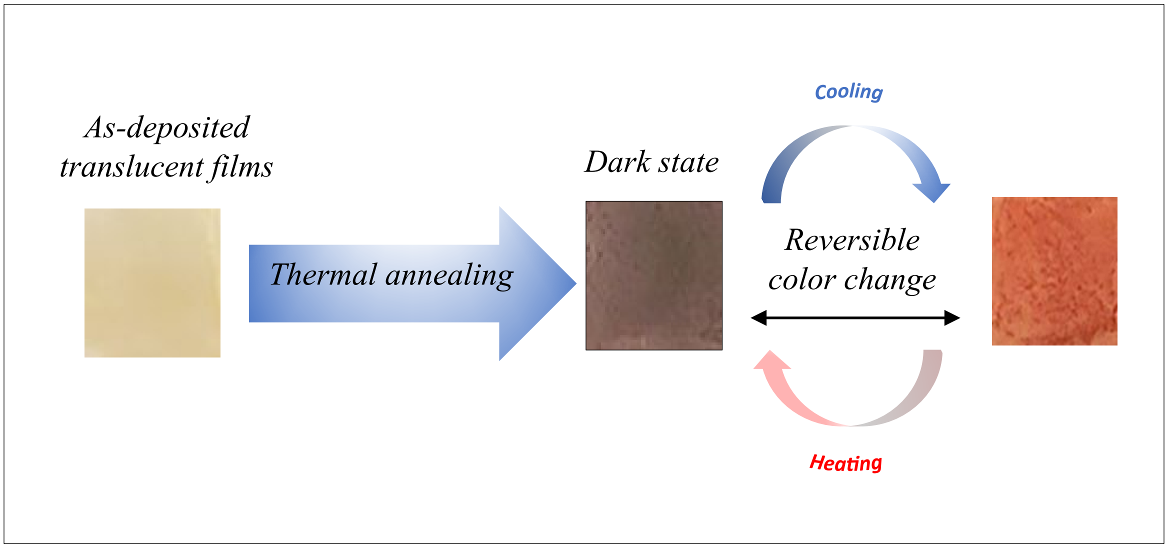

Figure S8. Moisture-induced discoloration in $NiCl_2$-based films before and after the dark thermal anneal test.

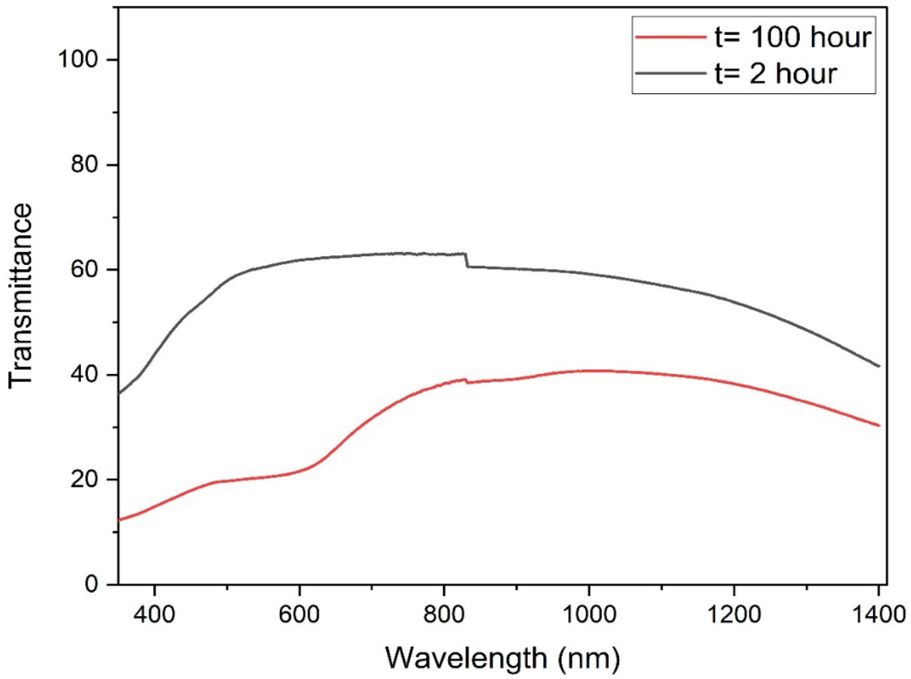

Figure S9. Temperature-dependent transmittance in $NiCl_2$-based films.
